# Combining a noble gas with radiotherapy: glutamate receptor antagonist xenon may act as a radiosensitizer in glioblastoma

**DOI:** 10.1186/s13014-023-02395-1

**Published:** 2024-01-30

**Authors:** Thomas Büttner, Marielena K. E. Maerevoet, Frank A. Giordano, Marlon R. Veldwijk, Carsten Herskind, Arne Mathias Ruder

**Affiliations:** 1 Department of Radiation Oncology, Medical Faculty Mannheim, University Medical Centre Mannheim, Heidelberg University, Theodor-Kutzer-Ufer 1-3, 68167 Mannheim, Germany; 2 Clinic for Urology and Paediatric Urology, University Hospital Bonn, Venusberg Campus 1, 53127 Bonn, Germany

**Keywords:** Glioma, Glioblastoma, Glutamate receptors, Xenon, Memantine, NMDA, AMPA, Radiotherapy, Radiation sensitizer, Clonogenic assay

## Abstract

**Background:**

Ionotropic glutamate receptors α-amino-3-hydroxy-5-methyl-4-isoxazole propionic acid receptor (AMPAR) and N-methyl-D-aspartate receptor (NMDAR) modulate proliferation, invasion and radioresistance in glioblastoma (GB). Pharmacological targeting is difficult as many in vitro-effective agents are not suitable for in patient applications. We aimed to develop a method to test the well tolerated AMPAR- and NMDAR-antagonist xenon gas as a radiosensitizer in GB.

**Methods:**

We designed a diffusion-based system to perform the colony formation assay (CFA), the radiobiological gold standard, under xenon exposure. Stable and reproducible gas atmosphere was validated with oxygen and carbon dioxide as tracer gases. After checking for AMPAR and NMDAR expression via immunofluorescence staining we performed the CFA with the glioblastoma cell lines U87 and U251 as well as the non-glioblastoma derived cell line HeLa. Xenon was applied after irradiation and additionally tested in combination with NMDAR antagonist memantine.

**Results:**

The gas exposure system proved compatible with the CFA and resulted in a stable atmosphere of 50% xenon. Indications for the presence of glutamate receptor subunits were present in glioblastoma-derived and HeLa cells. Significantly reduced clonogenic survival by xenon was shown in U87 and U251 at irradiation doses of 4–8 Gy and 2, 6 and 8 Gy, respectively (*p <* 0.05). Clonogenic survival was further reduced by the addition of memantine, showing a significant effect at 2–8 Gy for both glioblastoma cell lines (*p <* 0.05). Xenon did not significantly reduce the surviving fraction of HeLa cells until a radiation dose of 8 Gy.

**Conclusion:**

The developed system allows for testing of gaseous agents with CFA. As a proof of concept, we have, for the first time, unveiled indications of radiosensitizing properties of xenon gas in glioblastoma.

**Supplementary Information:**

The online version contains supplementary material available at 10.1186/s13014-023-02395-1.

## Background

A complex system promoting glioma invasion and growth has been recently discovered in several studies: By forming synapses with astrocytes, glioma cells receive proliferative stimuli that are passed on through tumor cell networks as depolarizing currents [1–5]. The excitatory neurotransmitter glutamate and ionotropic α-amino-3-hydroxy-5-methyl-4-isoxazole propionic acid receptors (AMPAR) play a key role in neuron–glioma interaction, where glioma cells receive glutamate through unidirectional synaptic connections [1, 3, 5]. In addition, glioma cells have been shown to secret large amounts of glutamate in their surroundings acting as an autocrine growth stimulant through AMPARs and N-methyl-D-aspartate receptors (NMDAR), as well as exhibiting toxic effects on adjacent brain tissue and, thus, facilitating tumor invasion [6–11].

In patients suffering from glioblastoma (GB), chemotherapy with alkylating agents has only a limited effect on tumor progression and the exploration of alternative treatment pathways is imperative [12, 13]. In light of this, pharmacological targeting of glutamate receptor pathways in GB has been studied, yet, most of the promising in vitro tested agents are not suitable for therapeutic usage in humans due to toxicity and several administrable substances have failed in vivo, possibly because of insufficient accumulation in brain tissue [5, 6, 11, 14–22].

Xenon gas is an established AMPAR and NMDAR antagonist for anaesthesia [23, 24]. It is a known neuroprotectant approved for the treatment of perinatal asphyxia and currently under investigation for application in patients after a circulatory arrest [25, 26]. As it accumulates in the central nervous system while being well tolerated, xenon is a promising glutamate receptor antagonist for GB therapy.

To enhance xenon’s potential, a combination with radiation therapy (RT) appears sensible as NMDAR antagonists together with ionizing irradiation have been shown to exhibit radiosensitizing effects in glioma in vitro [16, 27].

In radiobiological research, clonogenic survival as determined using the colony formation assay (CFA) represents an established method to evaluate the in vitro effects of RT alone or in combination with additional substances [28]. While elements, molecules or pharmaceutical agents in gaseous form (e.g. oxygen (O_2_), nitrogen (N_2_), nitrous oxide or volatile anaesthetics) can alter radiation effects on cells and therefore exhibit modulating properties in the CFA, their application is more challenging compared to liquids or solids simply dissolved into the cell culture growth medium [29–34].

In the past, the development of methods altering cell culture atmospheric conditions in radiobiological research has mainly been driven by investigations of irradiation and hypo- or hyperoxic conditions, where gas exposure is most commonly performed by purging a chamber containing the cell culture via in- and outlet-valves [35–39]. Literature indicates only few studies that have investigated the impact of RT in combination with noble gases (e.g. helium, xenon, or krypton) on tumor cells [40, 41]. Despite providing first evidence for gas-mediated radiosensitization, further studies are missing. A reason might be the lack of elaborate methods for gas exposure.

We designed and validated a system to conduct CFAs with gas exposure before, during or after RT and, for the first time, investigated the radiosensitizing effect of xenon gas (Xe) exposure on GB cells.

## Methods

### Vented cap T25 flasks

T25 flasks (FALCON® 25 cm² Rectangular Canted Neck Cell Culture Flask with Vented Cap, Corning Inc., Corning, USA) contain liquid cell medium and residual air/gas, with a total capacity of 70.0 ml (FALCON® technical data sheet). Additionally, the volume displaced by a capped T25 was determined via submersion in water to be 96.0 ml. The flasks’ caps allow for gas exchange with a hydrophobic membrane consisting of polytetrafluoroethylene (PTFE) with pores of 0.2 μm.

### Gas exposure

Our gas exposure system consists of a chamber made from acrylic glass with inner dimensions of 15.0 × 18.0 × 14.0 cm defining a volume of 3780.0 cm^3^ and a detachable lid containing a round hole (Ø 3.0 cm). Designed to be used in combination with vented cap T25 cell culture flasks, up to 15 flasks can be stockpiled inside. First, a polymethylene (PM) bag is placed into the chamber with its open side facing the upwards and flasks are inserted. The open side of the PM-bag is then fitted through the hole in the lid and connected to the ending of a flexible silicon tube in a gas-tight manner. The lid is attached and a vacuum pump (Mityvac® Silverline No. 04010, Lincoln Industrial, St. Louis, USA) is attached to the gas tubing, subsequently removing air until the PE-bag is empty and abuts the stack of T25 flasks. Now, vessels containing prepared gas mixtures can be connected to the gas tubing and the PE-bag can be refilled until its expansion reaches the chambers inside limits, thus defining an exact volume of the filling. Simple clamping of the gas tubing afterwards preserves the atmosphere inside (Supplementary Fig. 1).

### Validation

Stability of gas levels was tested by monitoring O_2_ as a tracer gas with an O_2_-sensor (GOX 100, GHM Group Greisinger GmbH, Regenstauf, Germany) inside the PE-bag during exposure with 100% O_2_ (Nippon Gases Deutschland GmbH, Düsseldorf, Germany).

Redistribution of gases within the flasks was examined with O_2_ and carbon dioxide (CO_2_) as tracers. According to the exposure method described above, T25 flasks with vented caps were exposed to 100% O_2_ with the O_2_ sensor inside one of the flasks. Additionally, diffusion of larger molecules through the vented cap was tested by exposing a flask to an atmosphere comprised of 5% CO_2_ in ambient air and tracking CO_2_ inside the flask with a CO_2_ sensor (GM70, Vaisala Oyj, Vantaa, Finland).

Lastly, dose aberration by Xe inside T25 flasks was measured. With an ionization chamber mounted inside a flask, a Xe-containing atmosphere (50% Xe, 25% N_2_, 20% O_2_, and 5% CO_2_) was established in the flask as described above. Then, irradiation was performed with the measurement flask and the surrounding dummy flasks between acrylic glass plates to balance secondary electrons from photons of 6 MV energy (Elekta Synergy, Elekta AB, Stockholm, Sweden).

### Cell culture

U87 and U251 were selected as established cell lines in glioblastoma models that have been tested in previous glutamate-receptor targeted radiobiology studies [16]. HeLa cells, derived from squamous cell carcinoma, were chosen as non-glial derived control. Cells were purchased from Cell Lines Service GmbH (CLS, Eppelheim, Germany), and confirmed for identity by short tandem repeat (STR) typing (service provided by CLS). Cells were cultivated in Dulbecco’s modified eagle’s medium (DMEM, Biochrom AG, Berlin, Germany) supplemented with 10% fetal bovine serum (GE Healthcare, Chicago, USA) and stored in a 37° C incubator gassed with additional 5% CO_2_. Cells were subcultured regularly before confluence and maintained at low passage numbers (< 10).

### Colony formation assay

The CFA was performed in triplicates of T25-flasks for each irradiation dose of 0, 2, 4, 6 and 8 Gy in three independent experiments. In preparation of CFA, cells were harvested with trypsine (Biochrom AG) and EDTA (1:3, Carl Roth GmbH, Karlsruhe, Germany), counted with a Neubauer counting chamber, and distributed into T25 flasks starting with 100 (U251), 200 (HeLa) or 1000 cells per flask (U87) for non-irradiated groups increasing the cell count with irradiation dose to 200, 600, 800, 2000 (U251), 400, 800, 2000, 4000 (HeLa) or 1000, 2000, 5000, 10,000 (U87). In a pretreatment plating setting, cells were allowed to attach overnight and either 25 µM of memantine-hydrochloride 0.25 mg/ml (Sigma-Aldrich Corp., St. Louis, USA) or distilled water were added one hour prior to irradiation.

#### Irradiation

For irradiation, T25 flasks triplicates were placed on top of of eight acrylic glass plates (thickness 1.0 cm) with two plates further added on top for buildup of secondary electrons (Supplementary Fig. 2). Vertical gantry position of a conventional linear accelerator (Elekta Synergy, Elekta AB, Stockholm, Sweden) was allocated by wall-mounted lasers marking the centre of the flasks’ caps membrane, horizontal position by opening the linear accelerators light field (32.0 × 32.0 cm) and positioning the plates centrally. The triplicate from the 0 Gy dose group was removed immediately afterwards and exchanged with dummy T25-flasks filled with 5 ml of water. After delivery of 6 MV photons, equivalent to an effective dose of 2 Gy, the 2 Gy triplicate was removed and replaced by dummy flasks. This process was repeated until a cumulative dose of 8 Gy for the last triplicate was reached.

#### Gas exposure

Gas exposure was performed immediately after irradiation. The flasks were exposed to a preformed gas mixture for 60 min comprised of either 75% Xe, 20% O_2_ and 5% CO_2_ (Air Liquide S.A., Paris, France) or 75% N_2_, 20% O_2_ and 5% CO_2_ (Nippon Gases Deutschland GmbH, Düsseldorf, Germany). This resulted in an atmosphere inside of the flasks of either 50% Xe, 25% N_2_, 20% O_2_, and 5% CO_2_ for xenon groups or 75% N_2_, 20% O_2_, and 5% CO_2_ for memantine or control groups. After exposure, the flasks’ vented caps were sealed and stored at 37° C for 24 h. Finally, the vented caps were unsealed and flasks were transferred into the 5% CO_2_-incubator.

#### Evaluation

Eleven (U251, HeLa) or 14 (U87) days after irradiation and gas exposure, cells were fixed with methanol and acetic acid (3:1), stained with crystal violet (all obtained from Carl Roth GmbH) and colonies of more than 50 clonal cells were counted. Surviving fraction (SF) was calculated and normalized to the respective plating efficiencies, given the prior observation of an approximately linear relationship between seeded cells and colonies counted [42]. and fitted by the linear-quadratic model (ln(SF) = -(αD + βD²)) by nonlinear regression using SigmaPlot v11 (SigmaPlot, Systat Software Inc., San Jose, USA).

### Indirect immunofluorescence staining

A method for glutamate receptor staining was performed as described before [43]. Cells were harvested and counted as described above. U87, U251, or HeLa cells were transferred onto an 8-well chamber slide (FALCON® 8 well cultureslide, Corning Inc., Corning, USA) with 6 × 10^4^ cells per well and allowed to attach overnight. Fixation was started with 200 µl 3.7% paraformaldehyde (Sigma-Aldrich Corp.) for 10 min followed by 200 µl 1% bovine serum albumin (Carl Roth AG) for 20 min. Next, 200 µl of rabbit-derived primary antibodies GluN1 (Ab109182, Abcam, Cambridge, UK), in 1:50 dilution and GluA1 (13,185 S, Cell Signaling Technology, Cambridge, UK)) in 1:200 dilution were added in one well each. Again, cells were incubated overnight. Afterwards, secondary antibody goat anti-rabbit IgG Fluor (AP307F, Merck KGaA, Darmstadt, Germany) was added in 1:200 dilution. Each step was succeeded by application of phosphate buffer saline (PBS; Thermo Fisher Scientific, Waltham, US). All antibody dilutions were achieved by BSA-PBS. After one hour of darkened and refrigerated storage, VECTASHIELD® mounting medium with 4′,6-diamidino-2-phenylindole (DAPI; Vector Laboratories Inc., Newark, US) was added and results were assessed with fluorescence microscopy.

### Statistical analysis

All experiments, except for immunostaining, were repeated at least three times. Mean, standard deviation, standard error and 95% confidence interval were calculated unless otherwise mentioned. An unpaired, two-tailed t-test was used to test statistical significance (p < 0.05).

## Results

### Gas exposure method

#### Handling characteristics

Our gas exposure technique offered simple handling using readily available and cost-effective components. The timing of exposure could be scheduled either before or after irradiation, providing flexibility. The amount of gas consumed depended on factors such as the desired concentration, the type of gas mixture used, and the quantity and type of cell culture containers employed. In our specific setup, we required 2340 ml of gas mixture for every 15 flasks used. Importantly, our experiments did not reveal any instances of microbiological contamination.

#### Validation

The PE-bag filled with pure oxygen (100%) exhibited satisfactory stability of oxygen levels during gas exposure, showing a minor reduction in oxygen concentration to 96.8% (with a range of 96.7–97%) within a 60-minute timeframe. Under exposure conditions, the final gas concentration inside of the 15 flasks can be calculated assuming an ideal gas distribution:

$$ \frac{V\left(flasks\right)*c\left(flasks\right)+V\left(bag\right)*c\left(gas\,mixture\right)}{V\left(chamber\right)}$$

In our specific validation scenario, this led to a calculated oxygen concentration of 81%. Following 60 min of exposure, we measured an average oxygen concentration of 80.6% (with a range from 79.8 to 81.2%), closely aligning with our calculated values and indicating an almost ideal distribution of gases.

Furthermore, we observed an increase in carbon dioxide (CO_2_) concentration inside the flasks, reaching 3.246% after 60 min. This demonstrated the effective passage of molecules larger than oxygen through thePTFE membranes of the caps.

When the flasks filled with xenon (Xe) were subjected to irradiation, there was a notable alteration in the effective radiation dosage. Specifically, it amounted to 2022 mGy (with a 95% confidence interval of [2020.6-2023.4]), in contrast to ambient air, which resulted in a dosage of 1993 mGy (with a 95% confidence interval of [1990.5-1995.4]).

### Clonogenic survival

#### Plating efficiency

**Fig. 1:**
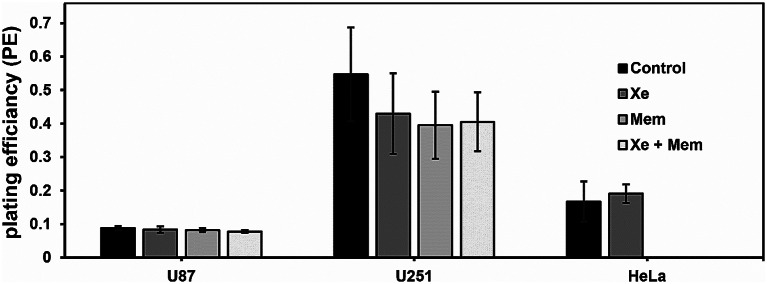
Plating efficiency (PE) of U87, U251 and HeLa cell lines treated with Xenon (Xe), Memantine (Mem) or both. Error bars indicate 95% confidence interval. No significant PE differences between treatments were observed

The plating efficiency of the sham-irradiated U87, U251 and HeLa cells by treatment groups Xe, Mem, Xe + Mem and controls is displayed in Fig. 1. There were no significant intergroup differences, suggesting no significant intrinsic toxicity of the agents.

#### Surviving fraction

**Fig. 2:**
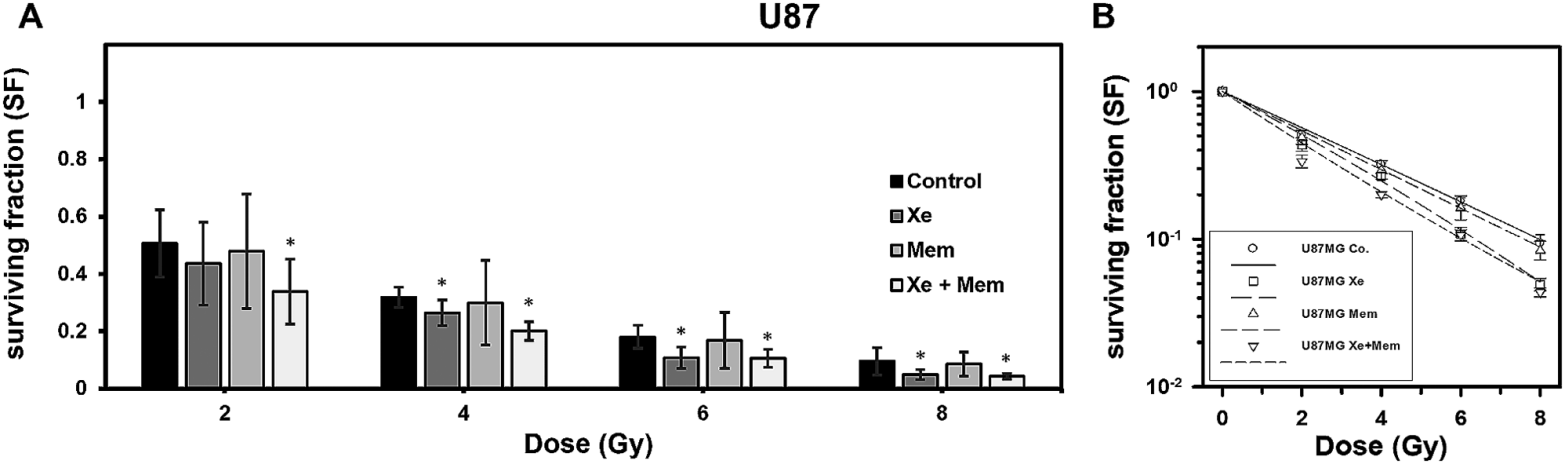
(**A**): Surviving fraction (SF) of U87 cells irradiated with 2–8 Gy and treated with xenon (Xe), memantine (Mem) or both (Xe + Mem). Error bars indicate 95% confidence interval. Significant reduction in SF was found for Xe starting at a dose of 4 Gy and for Xe + Mem starting at 2 Gy (*p < 0.05). (**B**): Linear-quadratic regression analysis. Error bars indicate standard error of mean. Data are given as means of at least three independent experiments and were normalised to plating efficiency. Non-normalized data are shown in the appendix (Supplementary Fig. 3)

Following irradiation of U87 cells, significant alterations in surviving fraction (SF) were observed in cells exposed to Xe at radiation doses of 4 Gy (p = 0.029), 6 Gy (p = 0.010), and 8 Gy (p = 0.037). Memantine alone did not result in statistically significant reduction of the mean SF compared to the control group. However, when both Xe exposure and memantine were combined, significant differences in SF were observed at radiation doses of 2 Gy (p = 0.023), 4 Gy (p = 0.001), 6 Gy (p = 0.008), and 8 Gy (p = 0.022). A summary of these results can be found in Fig. 2, with additional data provided in the appendix 1.

**Fig. 3:**
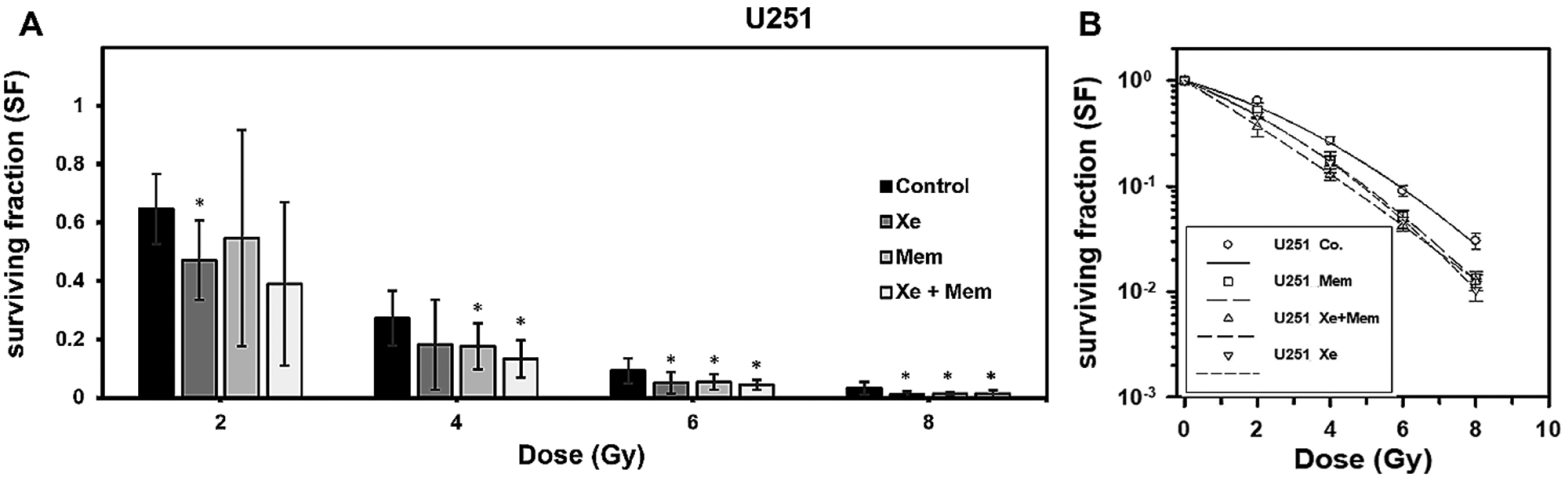
(**A**): Surviving fraction (SF) of U251 cells irradiated with 2–8 Gy and treated with xenon (Xe), memantine (Mem) or both (Xe + Mem). Error bars indicate 95% confidence interval. Significant reduction in SF was found for Mem or Xe starting at a dose of 4 Gy and for Xe + Mem starting at 2 Gy (*t-test p < 0.05). (**B**): Linear-quadratic regression analysis. Error bars indicate standard error of mean. Data are given as means of at least three independent experiments and were normalised to plating efficiency. Non-normalised data are shown in the appendix (Supplementary Fig. 3)

Subsequent to irradiation of 251 cells, significant differences in SF were found in cells exposed to Xe at radiation doses of 2 Gy (p = 0.009), 6 Gy (p = 0.027), and 8 Gy (p = 0.016). Similarly, memantine exhibited a significant reduction in SF at radiation doses of 4 Gy (p = 0.021), 6 Gy (p = 0.023), and 8 Gy (p = 0.019). Likewise, the combination of Xe and memantine yielded conspicuous differences in SF at radiation doses of 2 Gy (p < 0.017), 4 Gy (p < 0.003), 6 Gy (p < 0.006), and 8 Gy (p < 0.026). An overview of these outcomes is displayed in Fig. 3, while additional data can be found in the appendix 1.

**Fig. 4:**
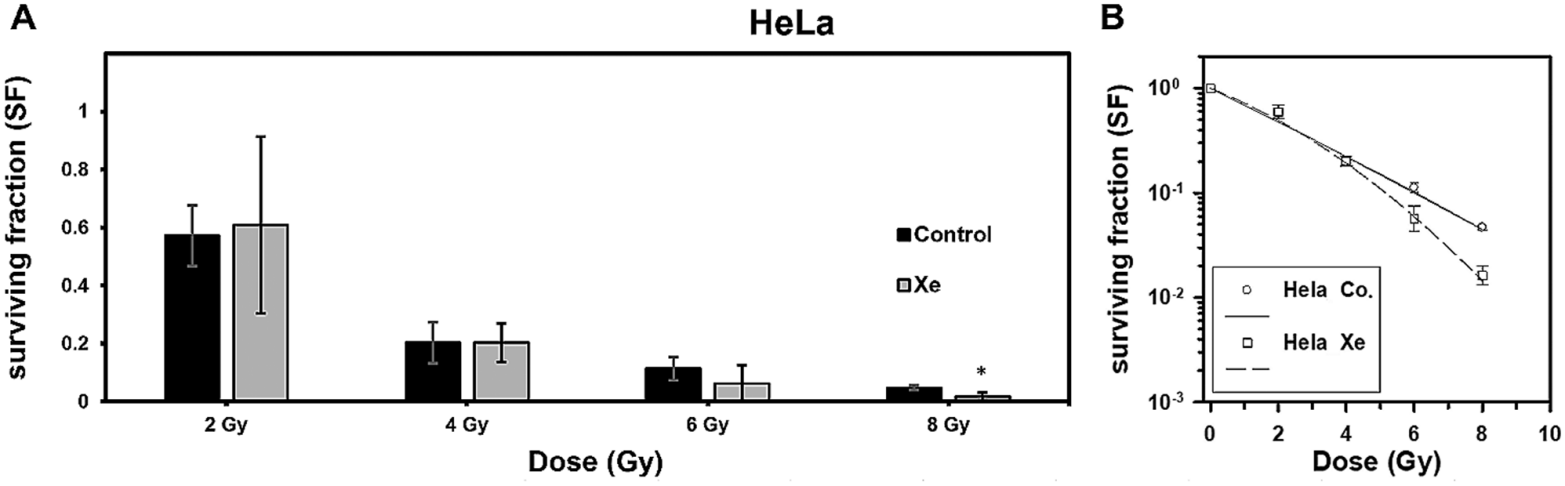
(**A**): Surviving fraction (SF) of HeLa cells irradiated with 2–8 Gy and treated with xenon (Xe). Error bars indicate 95% confidence interval. Significant reduction in SF was found for Xe at a dose of 8 Gy (*t-test p < 0.05). (**B**): Linear-quadratic regression analysis. Error bars indicate standard error of mean. Data are given as means of at least three independent experiments and were normalised to plating efficiency. Non-normalised data are shown in the appendix (Supplementary Fig. 3)

For non-glia derived HeLa, following irradiation, noteworthy differences in survival fraction (SF) were evident in cells treated with Xe at a radiation dose of 8 Gy (p = 0.029). Findings are presented in Fig. 4, additional data can be found in the appendix 1.

### AMPAR and NMDAR expression

**Fig. 5:**
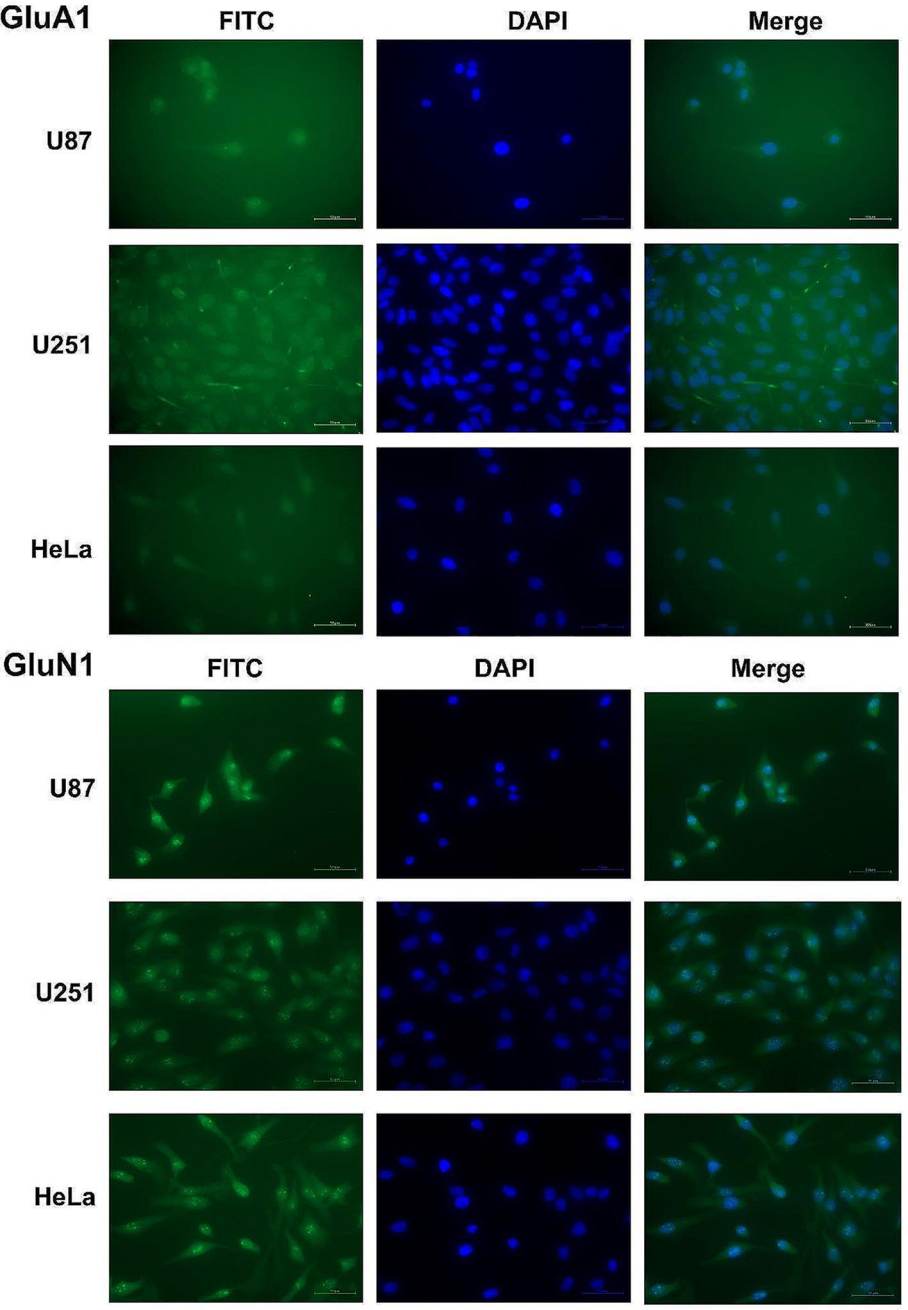
Immunofluorescence staining of U87, U251 and HeLa cells using N-methyl-D-aspartate receptor (NMDAR) subunit GLUN1 antibodies as well as α-amino-3-hydroxy-5-methyl-4-isoxazole propionic acid receptor (AMPAR) subunit GLUA1 antibodies (flouresceine green coloured) merged with 4′,6-diamidino-2-phenylindole (DAPI) for nuclear staining (blue coloured). As all cell lines showed staining of GluN1 and GluA1, we consider this as an indication for the presence of NMDAR and AMPAR

Figure 5 showcases a selection of immunofluorescence microscopy images that offer a visual representation of our findings. Notably, these images reveal the presence of signals corresponding to the receptor subunits GluA1 (AMPAR) and GluN1 (NMDAR) in U87, U251 and HeLa cell lines. We consider these indications, sufficient to sustain the hypothesis of a glutamate receptor-mediated effect in clonogenic survival.

## Discussion

Despite intensive research efforts, glioblastoma remains an often fatal disease with limited therapeutic options [44]. Whereas targeted therapies have achieved relevant advances in numerous entities, this approach does not yet seem to be successful in GB and there is an urgent need for more effective treatments to improve outcomes [13, 45].

Targeting ionotropic glutamate receptors to reduce proliferation and invasion of GB, promising preclinical data exist *inter-alia* for AMPAR- and NMDAR-inhibitors including memantine [3, 6, 8, 11, 15, 16, 20, 46]. As radiotherapy plays a central role in GB treatment, testing for radiosensitizing potential of glutamate receptor targeted therapy to enhance irradiation’s effectiveness appears sensible especially from a clinical perspective. In vitro data have, so far, shown radiosensitizing potential for the NMDAR-inhibitors ifenprodil, dizocilpine, riluzole and memantine [14, 16].

However, transfer to clinical application is difficult as unacceptable toxicity or bioavailability in the central nervous system (CNS) can be limiting factors [47]. Although the blood-brain barrier (BBB) is considered locally disrupted in GB, the disease occultly infiltrates the brain tissue [5, 48]. Therefore, therapeutic agents must be also capable of targeting tumor cells located in areas with an intact BBB. A further essential characteristic of a useful agent is adequate tolerability at therapeutic doses, which, due to the frequently occurring and accumulating side effects of surgery plus irradiation, is a major concern for GB patients [49, 50]. Here, the AMPAR- and NMDAR-antagonist xenon represents a long-tested and approved drug, which readily crosses the blood-brain barrier [51, 52]. Due to its favourable toxicity profile good tolerability in patients is expected even at high concentrations of up to 50% xenon [53]. To explore whether xenon does exhibit anti-tumoral and, possibly, radiosensitizing effects, we have designed and validated a method for combining xenon gas exposure with a conventional CFA, the gold standard for determining effects on cellular clonogenic survival.

As trace gas in the atmosphere, commercial extraction of xenon is expensive [54]. The entry into our research was thus marked by the lack of a method that would allow for an economical use of gaseous agents while being compatible with the requirements of a CFA. Hence, as other authors have previously experienced, we were compelled to develop a proprietary system [39].With our method, we were able to reduce xenon consumption to about a quarter of the amount required by purging with inlet and outlet valves [55]. In the subsequent validation experiments, we were able to maintain a stable gas atmosphere in the exposure system and found a gas distribution that showed adequate approximation to our calculated target values. Since the physical behaviour of the larger xenon molecules cannot be fully depicted by O_2_, gas distribution was additionally investigated using CO_2_. Carbon dioxide resembles xenon in collision diameter and Boltzmann constant as the relevant parameters according to Chapman-Enskog theory while xenon, vice versa, is used as an established tracer gas for CO_2_ [56, 57]. Therefore, we consider our CO_2_ diffusion measurements across the PTFE membrane to be applicable for xenon. Detecting a significant radiation dose increase due to the presence of high-Z element xenon within the T25 flasks resembled qualitative evidence of the altered atmospheric composition and validated our approach for the subsequent cell culture experiments.

As a subsequent proof of concept, we could show for the first time that xenon applied after irradiation seems to mediate a radiosensitizing effect on AMPAR- and/or NMDAR-positive glioblastoma cell lines U87 and U251 in the CFA. The observed radiosensitizing effect was further enhanced by the addition of memantine, while a stand-alone effect of memantine has already been described before and was observed again in our study [16].

Previous research has revealed that NMDAR-mediated calcium influx with downstream signalling pathways represents a key factor in repairing irradiation-induced double-strand-breaks and accounts for radiation resistance in GB [16]. Presumably, the results obtained demonstrate impaired DNA repair after irradiation mediated by the inhibition of the calcium permeable glutamate receptors. Memantine inhibits calcium influx through NMDAR, whereas xenon modulates calcium permeability of NMDAR as well as AMPAR [58, 59]. The hypothesis is supported by the low impact of xenon, memantine, or a combination of both on cell survival in the sham-irradiated groups lacking irradiation-induced DNA damage. Because xenon and memantine act at different binding sites at the NMDAR, namely the glycine binding site for xenon and the Mg^2+^ binding site for memantine, there is no competitive but possibly an additive effect when both substances are coupled [60]. The enhanced radiosensitizing effect observed in groups treated with both substances may consort with a twofold receptor modulation. Our hypothesis is further corroborated by immunofluorescence staining, demonstrating expression of the corresponding receptors subunits in both cell lines as described in earlier reports [8, 9, 27, 61]. The interpretation of the immunofluorescence staining is limited, as it allows for a qualitative NMDAR/AMPAR indication but methodically lacks quantitative glutamate receptor assessment and comparison between cell lines. These analyses would require a western blot or quantitative real-time polymerase chain reaction.

Overall, however, the mechanism of action of xenon in GB has not been completely understood and it cannot be ruled out whether additional signalling pathways are involved, yet. The detection of a considerably weaker radiosensitization of HeLa cells at high irradiation doses indicates that glutamate receptors might exhibit different downstream effects in non-glial cell lines.

Our preliminary testing aimed to explore xenon’s potential as glutamate receptor antagonist and the gas was therefore applied only after irradiation. Effective radiation dose was altered when irradiation was delivered under xenon gas atmosphere, likely because of xenon’s high atomic number. As an altered effective radiation dose not only affects tumor cells but also surrounding healthy tissue possibly increasing side effects in a clinical setting, xenon gas use during irradiation does not seem feasible. Yet, the immediate application of xenon after irradiation would be desirable as DNA damage repair in tumor cells starts within minutes after DNA damage is inflicted [62].

### Limitations and prospects

Several limitations restrict the conclusions of our work. First and foremost, the study was conducted with two-dimensional (2D) culture in vitro and its effects need to be confirmed in more suitable models such as three-dimensional (3D) in vitro culture and in vivo. Furthermore, even with very robust results, the investigation was carried out on no more than two glioblastoma cell lines. We want to point out that among these, U87 was reported to be potentially misclassified, although extensively used in GB research [63]. The method of PE-based normalisation of surviving fraction has been reported to potentially generate assay-intrinsic errors due to cellular cooperation [42]. Reactive nitrogen species (RNS) are involved in the effects of ionizing radiation and by reducing the nitrogen fraction in the xenon groups bias can be created. However, RNS formation is transient and, thus, presumably already completed at the time of atmospheric alterations, furthermore, a relationship of RNS to ambient nitrogen concentration so far has not been described [64]. Our method of gas exposure allows for an economical and simple use of the expensive noble gas xenon but has not yet been described before and can potentially lead to biased results. Despite its limitations, our data provide a strong rationale for further exploration. Unlike other substances that have demonstrated radiosensitization of glioblastoma cell lines, xenon and memantine are easily available and approved substances that have few side effects and thus allow for an early transfer into a preliminary clinical setting.

## Conclusion

In summary, the ionotropic glutamate receptor pathway in GB that recently moved into the centre of research continues to be a promising target for new treatment options. With our work, we provide a novel method to investigate the impact of gaseous agents in colony forming assay. Furthermore, we gathered the first evidence suggesting xenon, an already approved drug with excellent CNS accumulation a low toxicity profile, might harbour radiosensitizing properties in GB cell lines.

## Electronic Supplementary Material

Below is the link to the electronic supplementary material.

Supplementary Material 1 contains a presentation of the gas exposure technique and the irradiation setup. Furthermore, it shows the non-PE-normalized linear-quadratic regression analysis plots on surviving fraction and provides a tabular view of plating efficiency and surviving fraction in all cell lines, dose groups and treatments

## Data Availability

The datasets generated and analysed during the current study are available from the corresponding author on reasonable request.
