# Supplementary material for: Combining a noble gas with radiotherapy: glutamate receptor antagonist xenon may act as a radiosensitizer in glioblastoma
Source: Radiat Oncol. 2024 Jan 30;19:16. doi: 10.1186/s13014-023-02395-1 (PMC10826195; doi:10.1186/s13014-023-02395-1)
Supplement: Supplementary file 1 — Supplementary Material 1 contains a presentation of the gas exposure technique and the irradiation setup. Furthermore, it shows the non-PE-normalized linear-quadratic regression analysis plots on surviving fraction and provides a tabular view of plating efficiency and surviving fraction in all cell lines, dose groups and treatments [file 13014_2023_2395_MOESM1_ESM.docx]

**Appendix**


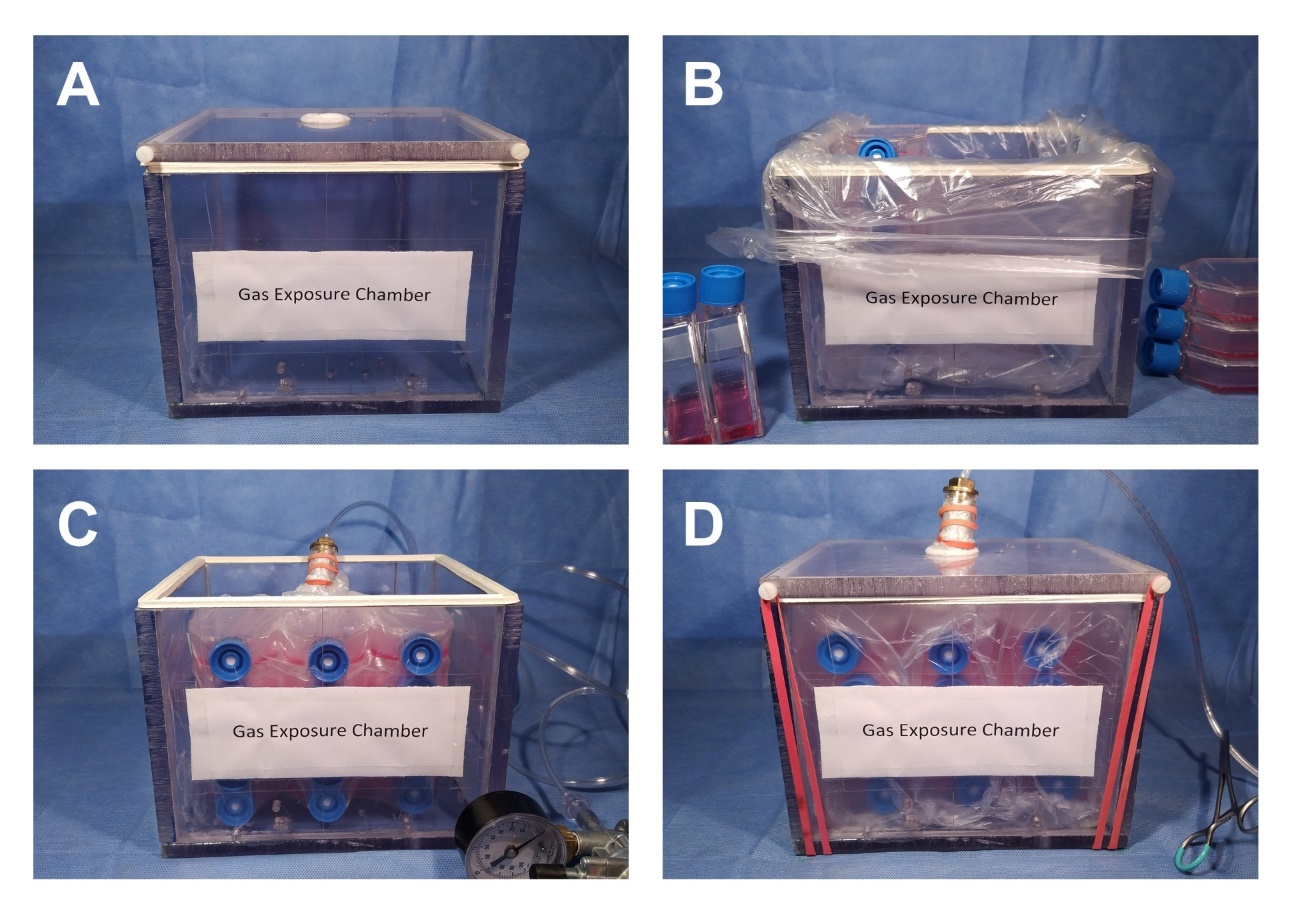


Supplementary Figure 1: Gas exposure setup. A: Empty chamber. B: Polymethylene (PM) bag applied and flasks are stocked inside. C: PM bag closed and attached to vacuum pump. D: Closed chamber with inflated PM Bag inside, clamped gas tubing.


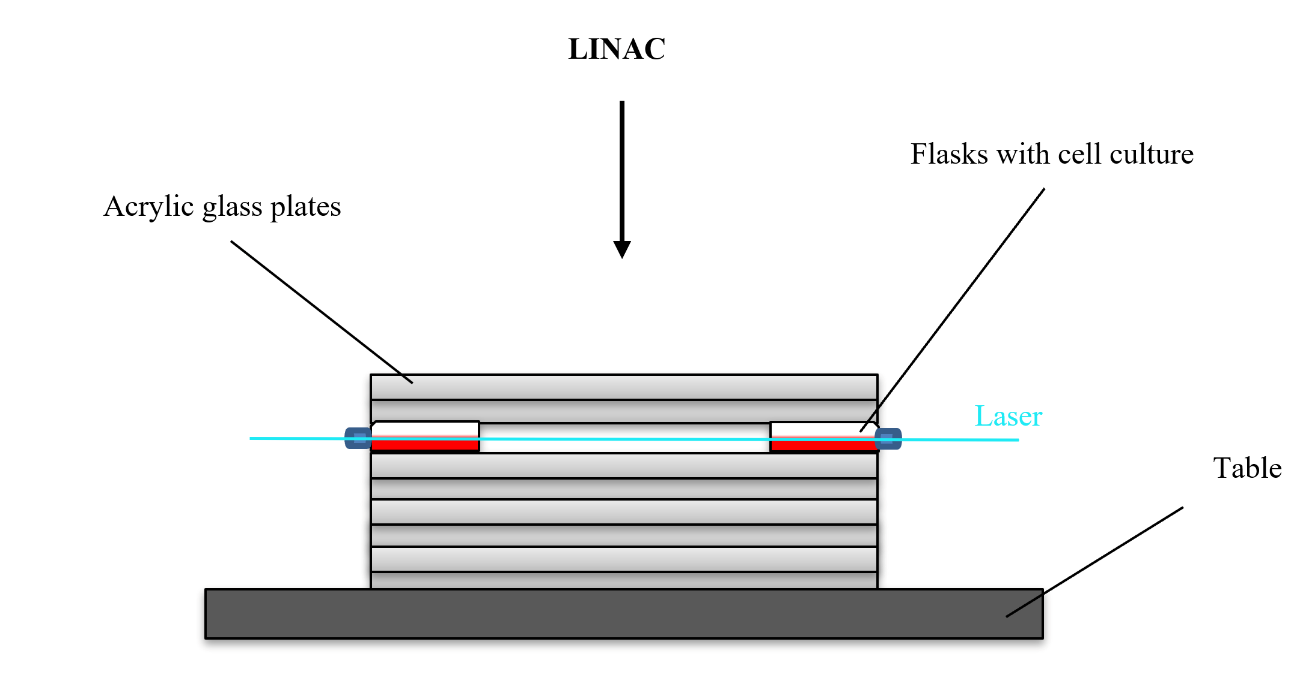


Supplementary Figure 2: Irradiation/Dosimetry setup: T25 flasks are placed between acrylic glass plates, the wall laser is used for alignment at the linear accelerator (LINAC).


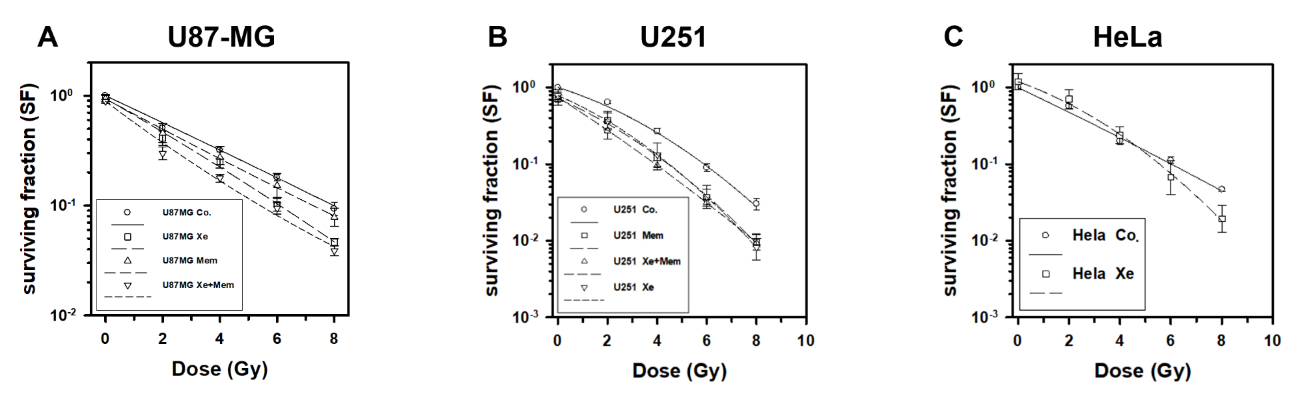


Supplementary Figure 3: Linear-quadratic (LQ) regression analysis of U87, U251 and HeLa cell lines surviving fraction (SF) without normalisation to plating efficiency. A: U87 cells. B: U251 cells. C: HeLa cells. Data are given as means of at least three independent experiments; error bars indicate standard error of mean.

|  | **Controls** | **Xe** | **Mem** | **Xe+Mem** |
| --- | --- | --- | --- | --- |
| **U87** |  |  |  |  |
| PE | 0.088 | 0.084 | 0.082 | 0.078 |
| SF (0 Gy) | 1.000 | 1.000 | 1.000 | 1.000 |
| SF (2 Gy)  95 % CI | 0.506  [0.391, 0.621] | 0.437  [0.295, 0,578] | 0.479  [0.283, 0.675] | **0.339***  **[0.227, 0.450]** |
| SF (4 Gy)  95 % CI | 0.319  [0.284, 0.352] | **0.264***  **[0.220, 0.307]** | 0.300  [0.155, 0.445] | **0.201****  **[0.169, 0.232]** |
| SF (6 Gy)  95 % CI | 0.180  [0.141, 0.220] | **0.108***  **[0.072, 0.145]** | 0.168  [0.072, 0.264] | **0.107****  **[0.076, 0.138]** |
| SF (8 Gy)  95 % CI | 0.095  [0.048, 0.142] | **0.049***  **[0.031, 0.067]** | 0.085  [0.045, 0.126] | **0.044***  **[0.039, 0.054]** |
| **U251** |  |  |  |  |
| PE | 0.547 | 0.430 | 0.395 | 0.405 |
| SF (0 Gy) | 1.000 | 1.000 | 1.000 | 1.000 |
| SF (2 Gy)  95 % CI | 0.646  [0.525, 0.766] | **0.471****  **[0.335, 0.607]** | 0.547  [0.177, 0.917] | **0.390***  **[0.110, 0.670]** |
| SF (4 Gy)  95 % CI | 0.272  [0.178, 0.366] | 0.181  [0.027, 0.335] | **0.175***  **[0.098, 0.253]** | **0.132****  **[0.068, 0.196]** |
| SF (6 Gy)  95 % CI | 0.092  [0.049, 0.135] | **0.050***  **[0.014, 0.087]** | **0.053***  **[0.027, 0.080]** | **0.043****  **[0.026, 0.060]** |
| SF (8 Gy)  95 % CI | 0.032  [0.010, 0.054] | **0.011***  **[0.002, 0.021]** | **0.014***  **[0.010, 0.017]** | **0.013***  **[0.003, 0.024]** |
| **HeLa** |  |  |  |  |
| PE | 0.167 | 0.191 |  |  |
| SF (0 Gy) | 1.000 | 1.000 |  |  |
| SF (2 Gy)  95 % CI | 0.572  [0.467, 0.677] | 0.608  [0304, 0.913] |  |  |
| SF (4 Gy)  95 % CI | 0.202  [0.131, 0.272] | 0.202  [0.135, 0.270] |  |  |
| SF (6 Gy)  95 % CI | 0.113  [0.073, 0.152] | 0.061  [0.000, 0.124] |  |  |
| SF (8 Gy)  95 % CI | 0.047  [0.038, 0.055] | **0.017****  **[0.004, 0.030]** |  |  |
| PE = plating efficiency, SF = surviving fraction, Xe = xenon, Mem = memantine, 95 % CI = 95 % confidence interval [lower limit, upper limit], t-Test p <0.05, **t-Test p <0.01 | | | | |
|  | | | | |

Supplementary Table 1: Plating efficiency (PE) and PE-normalised Surviving fraction of U87, U251 and HeLa cells treated with xenon (Xe), memantine (Mem) or both (Xe+Mem) in colony forming assay. Data are given as means and 95% confidence interval from 3 (U87, HeLa) or 4 (U251) independent experiments.
